# Peptide ^Tat(48–60)^ YVEEL protects against necrotizing enterocolitis through inhibition of toll-like receptor 4-mediated signaling in a phosphatidylinositol 3-kinase/AKT dependent manner

**DOI:** 10.3389/fnut.2022.992145

**Published:** 2022-10-10

**Authors:** Xiangyun Yan, Yan Cao, Wenjuan Chen, Qinlei Yu, Yanjie Chen, Shuwen Yao, Chengyao Jiang, Xiaohui Chen, Shuping Han

**Affiliations:** ^1^Department of Pediatrics, Nanjing Maternity and Child Health Care Hospital, Women’s Hospital of Nanjing Medical University, Nanjing, Jiangsu, China; ^2^Nanjing Maternity and Child Health Care Institute, Nanjing Maternity and Child Health Care Hospital, Women’s Hospital of Nanjing Medical University, Nanjing, Jiangsu, China

**Keywords:** necrotizing enterocolitis (NEC), enterocyte, peptide, TLR4, PI3K/AKT

## Abstract

Necrotizing enterocolitis (NEC) is a catastrophic disease largely occurring in preterm infants, and toll-like receptor 4 (TLR4) has been implicated in its pathogenesis. The current therapeutic strategies for NEC are, however, far from optimal. In the present study, a whey-derived antioxidative peptide conjugated with a cell-penetrating TAT [^Tat (48–60)^ YVEEL] was prepared to endow it with enhanced cell uptake capability and bioavailability. The protective effect of ^Tat (48–60)^ YVEEL on experimental NEC was evaluated both *in vitro* and *in vivo*. Inhibition of TLR4-mediated signaling by ^Tat (48–60)^ YVEEL was assessed in FHC and IEC-6 enterocytes, neonatal rat model of NEC, and the mechanism underlying this effect was determined. ^Tat (48–60)^ YVEEL significantly inhibited TLR4-mediated expression of pro-inflammatory cytokines, p65 nuclear translocation and restored the impaired enterocyte migration in cultured enterocytes. In addition, ^Tat (48–60)^ YVEEL administration strikingly increased the survival rate, and reduced the severity of NEC in rats through inhibition of TLR4-mediated signaling. These protective effects of ^Tat (48–60)^ YVEEL occurred in a PI3K/AKT dependent manner, as administration of PI3K activator Ys49 abrogated its protective effects. Combined with liposomes, ^Tat (48–60)^ YVEEL demonstrated longer retention in the intestines that better for potential clinical applications. These data demonstrate that ^Tat (48–60)^ YVEEL protects against NEC through inhibition of TLR4-mediated signaling in a PI3K/AKT dependent manner, and offer a potential therapeutic approach to this disease.

## Introduction

Necrotizing enterocolitis (NEC) is a serious and life-threatening intestinal disease that affects mostly the preterm infants (1), characterized by disruption of intestinal mucosa and bacterial/endotoxin (LPS) translocation across the inflamed intestine (2). Although the pathogenesis of NEC remains largely uncertain, several studies have identified the importance of the LPS receptor toll-like receptor 4 (TLR4) in its pathogenesis (3, 4). Activation of TLR4 in the intestinal epithelium leads to reduced epithelial restitution, increased villus loss and intestinal inflammation (5), and also contributes to the impaired intestinal perfusion resulting in intestinal necrosis which is a typical feature of NEC (6). It has been reported that mice with TLR4 deficient or received a TLR4 inhibitor are protected from NEC development (7, 8). Therefore, development of molecules that can limit TLR4-mediated signaling will hold a great promise as novel therapeutic agents for NEC.

Recent studies have demonstrated that bioactive components found in milk could be responsible for its protective effect against NEC in premature newborns (9, 10). For example, human milk oligosaccharides 2′-fucosyllactose (2′-FL) and 6′-sialyllactose (6′-SL) can reduce NEC by directly binding to TLR4 to inhibit the signaling transduction (11). Whey protein, such as lactoferrin, may be the important component responsible for the prevention of NEC (12), due to its antioxidant, antimicrobial, and immunomodulatory properties (13). Whey protein hydrolyzates exert anti-inflammatory effects on respiratory epithelial cells by a combination of decreased LPS binding to TLR4 and enhanced antioxidant ability (14). In seeking to understand the bioactive components of milk that could prevent NEC, we (15) and others (16) have shown that milk protein-derived peptides, chains of amino acids released from parent proteins, may be responsible for the protective benefits. Importantly, there has been no mechanism that links the potential benefits of these peptides against NEC.

In this study, we novelty generated a whey-derived antioxidative peptide (YVEEL) (17) conjugated with a powerful cell-penetrating peptide TAT (18) [^Tat (48–60)^ YVEEL], and investigated for the first time the role of ^Tat (48–60)^ YVEEL in the protection against experimental NEC both *in vitro* and *in vivo*. Our results demonstrated that ^Tat (48–60)^ YVEEL had a great inhibitory effect on TLR4-mediated signaling and may serve as a novel potential therapeutic candidate for NEC.

## Materials and methods

### Peptide synthesis

Peptide YVEEL and the HIV-1 TAT (48–60: GRKKRRQRRRPPQQ) (19)-hybrid YVEEL [^Tat(48−60)^ YVEEL], were synthesized by the solid-phase with the N1-9-fluorenylmethyloxycarbonyl (Fmoc) strategy. Peptide synthesis performed using HOBt esters of Fmoc-amino acids. The synthesis begins with activating the resin by swelling the resin and removing the protecting group. Piperidine (20%) was used to remove the Fmoc protecting group from the N-terminal amino acid residue and then obtain the resin-bound peptide. The peptide was cleaved from the applicable resins by treatment with a cleavage reagent. The purity of peptides was determined by high-performance liquid chromatography (HPLC) and exceeded 95% (Supplementary Figure 1). The peptides were dissolved in endotoxin-free water and stored at −80°C.

### Cell culture

The human normal colon epithelial cell (FHC) and rat small intestinal crypt cell line (IEC-6) were purchased from ATCC (Manassas, VA, USA). FHC cells were cultured in DMEM: F12 Medium (Gibco, USA), supplemented with fetal bovine serum (10%, Gibco, USA) and penicillin/streptomycin (1%, Gibco, USA), cholera toxin (10 ng/ml, Sigma-Aldrich, USA) and hydrocortisone (100 ng/ml, Sigma-Aldrich, USA). IEC-6 cells were cultured in DMEM medium (Gibco, USA), supplemented with fetal bovine serum (10%, Gibco, USA) and penicillin/streptomycin (1%, Gibco, USA). Cells were cultured at 37°C in a humidified 5% CO_2_ atmosphere, and the medium was changed every 2 days. Lipopolysaccharides (LPS, 50 μg/ml, Sigma-Aldrich, USA) was used to establish NEC *in vitro* cell model. ^Tat (48–60)^ YVEEL were pretreated at different concentrations (20 or 40 μM, respectively) for 1 h before LPS stimulation. In some experiments, PI3K inhibitor LY294002 (10 μM, MedChem, USA) or PI3K activator YS49 (2 and 5 μM, MedChem, USA) were used.

### Analysis of cellular uptake

The peptides [YVEEL or ^Tat (48–60)^ YVEEL] were labeled with fluorescein isothiocyanate (FITC) during the synthesis progress. A total of 1.5 × 10^5^ FHC or IEC-6 cells were seeded into 6-well plates. The FITC-YVEEL or FITC-^Tat(48–60)^ YVEEL were added to the medium of FHC or IEC-6 cells for 20, 40, and 60 min, respectively. After that, FHC or IEC-6 cells were washed three times with 1 × PBS, fixed in 4% paraformaldehyde (Biosharp, China), and stained with DAPI (Sigma-Aldrich, USA). Fluorescence signals were captured with a microscope (Zeiss, Germany).

### Western blot analysis

Cell pellets or intestinal tissues were lysed with RIPA buffer (Beyotime, China) for 20 min on ice and lysates were collected by centrifugation (13000 × *g*, 20 min at 4°C). Protein concentrations were quantified using the BCA Protein Assay (Thermo Fisher Scientific, USA) and equal amounts (20 μg) of protein lysates from each sample were separated by SDS-PAGE. Separated proteins were then transferred to PVDF membranes (Millipore, USA). Membranes were blocked with 5% skim milk or BSA and then incubated with the following primary antibodies: anti-TLR4 (Abcam, USA), anti-p-IκBα (Abcam, USA), anti-IκBα (Abcam, USA), anti-p-PI3K (Cell Signaling Technology, USA), anti-p-AKT (Cell Signaling Technology, USA), anti-PI3K (Cell Signaling Technology, USA), anti-AKT (Cell Signaling Technology, USA), anti-β-actin (Abcam, USA). Membranes were then incubated with species-matched HRP-conjugated secondary antibodies (Biosharp, China). Protein expressions were visualized using Immobilon^®^ Western Chemiluminescent HRP Substrate (Millipore, USA) in accordance with the manufacturer’s protocol.

### qRT-PCR analysis

Total RNA was extracted from cultured cells or intestinal tissues using Trizol Reagent (Invitrogen, USA). For examination of the mRNA expression, cDNA was synthesized from 1 μg of total RNA by using a Revert Aid first-strand cDNA synthesis kit (Takara, Japan). Then, quantitative reverse transcription polymerase chain reaction (qRT-PCR) analysis was performed with SYBR Premix Ex Taq (Takara, Japan) in ViiA 7 Real time system (Life technologies, USA). The sequences of primers were showed in Table 1. Data were calculated according to 2^–ΔΔCt^ method.

**TABLE 1:** Primers for qRT-PCR.

Target	Primer sequence (5′∼3′) (F: Forward; R: Reverse)
human-IL-6	F: TCTTCAGAACGAATTGACAAACAAA
	R: GCTGCTTTCACACATGTTACTCTTG
human-COX-2	F: CTGGCGCTCAGCCATACAG
	R: CGCACTTATACTGGTCAAATCCC
human-β-actin	F: TCCCTGGAGAAGAGCTACG
	R: GTAGTTTCGTGGATGCCACA
Rat-IL-6	F: AGCGATGATGCACTGTCAGA
	R: GGAACTCCAGAAGACCAGAGC
Rat-COX-2	F: ATCAGAACCGCATTGCCTCT
	R: GCCAGCAATCTGTCTGGTGA
Rat-β-actin	F: CACCCGCGAGTACAACCTTC
	R: CCCATACCCACCATCACACC

### *In vitro* enterocyte migration

A total of 3 × 10^5^ FHC or IEC-6 cells were seeded into 6-well plates. Cells were expected to reach a 100% confluence on the day of the experiment. Monolayers were scratched by a sterile pipette tip and washed for three times with pre-warmed 1 × PBS. The wounded monolayer of FHC or IEC-6 cells was imaged at indicated time using a microscope (Zeiss, Germany). The relative migration index of the cells was measured according to the area of the wound edge by Image J software (NIH Image, USA).

### Immunofluorescence staining

FHC cells were fixed, washed and blocked, and then incubated with the following primary antibodies: anti-NF-κB p65 (Cell Signaling Technology, USA) and DAPI (Sigma-Aldrich, USA). The Alexa Fluor^®^ 488-labeled goat anti-rabbit secondary antibody was used (Cell Signaling Technology, USA). FHC cells were counterstained with DAPI (Sigma-Aldrich, USA) and visualized using a fluorescence microscope (Zeiss, Germany).

### RNA extraction and high throughput sequencing

To further explore the underlying mechanisms of the protection functions of ^Tat (48–60)^ YVEEL, high throughput sequencings were used to identify differentially expressed mRNA profiles between LPS and LPS + ^Tat (48–60)^ YVEEL induced FHC cells. Total RNA was extracted using RNeasy Mini Kit (Qiagen, USA) according to the manufacturer’s protocols. RNA quality was assessed using the Agilent Bioanalyzer 2100 (Agilent technologies, USA). After RNA quality control, double-stranded cDNA was synthesized by reverse transcription and then cDNA was end-repaired. RT-PCR was applied for amplification and purification, followed with library screening by quality test. In brief, the whole transcriptome libraries were constructed using VAHTS Universal V6 RNA-seq Library Prep Kit for Illumina (Illumina, Vazyme, NR604-02) according to the manufacturer’s instructions. Bioinformatics analysis of the raw sequencing data was then carried out. Differentially expressed mRNAs screened by | fold change| >2.0 and *p*-value < 0.05 were considered statistically significant. Cluster Profiler R v3.8.1 and KOBAS v 3.0.3 were used for further Gene Ontology (GO) and KEGG pathway analysis.

### Animal experiments

This study was approved by the Institutional Review Board at Women’s Hospital of Nanjing Medical University, Nanjing Maternity and Child Health Care Hospital. Neonatal SD rats were randomly divided into 3 groups (15 rats per group) in the first 24 h after birth: Control (Ctrl), NEC and NEC + ^Tat (48–60)^ YVEEL. Experimental NEC was induced in newborn rats as previously described with modification (20). Briefly, rat pups were gavage fed with 50 μL g^–1^ body weight formula milk [15 g Similac 60/40 (Abbott Nutrition, USA) in 75 mL Esbilac (Pet-Ag, USA)] three times per day (8 ± 1 h intervals), as well as exposure to hypoxia (95% N_2_, 1 min) and cold stress (4°C, 10 min) twice a day for 4 days. ^Tat (48–60)^ YVEEL (10 mg/kg) was added to NEC formula with each gavage feeding three times daily for NEC + ^Tat (48–60)^ YVEEL Group. Pups in Ctrl Group was fed by their mothers. The number of deaths was recorded daily for all groups and calculated as the survival rate. The weight of each rat was also recorded every day. All pups were sacrificed at day 4. Intestinal samples were harvested and then fixed in 4% paraformaldehyde solution, embedded in paraffin, and analyzed with hematoxylin and eosin (H&E) staining and Immunohistochemical (IHC) analysis for microscopic evaluation. The severity of NEC was assessed using a previously established scoring system from 0 (normal) to 3 (severe) as previously published (21). To measure enterocyte migration *in vivo*, animals were injected with bromodeoxyuridine (BrdU) (50 mg/kg; Sigma-Aldrich, USA) 6 h before sacrifice, and tissues were immunostained for BrdU expression and visualized using microscopy (Zeiss, Germany). Enterocyte migration was determined by measuring the distance from the bottom of the crypt to the foremost-labeled enterocyte as described previously (22). The *in vivo* behavior of ^Tat (48–60)^ YVEEL in mice was determined by FITC-labeled ^Tat (48–60)^ YVEEL (50 μg/ml) with or without liposome (500 μg/ml) encapsulation (liposome: peptide ratio 10:1) at indicated hours post intragastric administration using *in vivo* imaging system (IVIS Spectrum, PerkinElmer, USA), and then the fluorescent intensity of each organ was evaluated, respectively.

### Data analysis

Statistical analysis was performed with GraphPad version 7.0. Data were analyzed by two-tailed Student’s *t*-test or ANOVA. A *p*-value of less than 0.05 was statistically significant.

## Results

### Cellular uptakes of peptide YVEEL with or without HIV-1 TAT (48–60)

**FIGURE 1:**
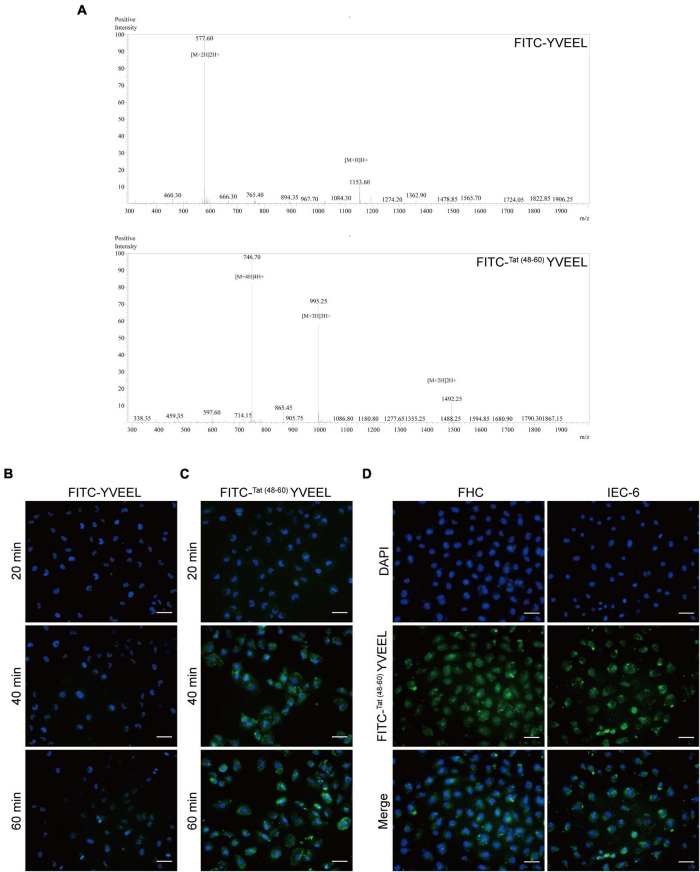
Cellular entry of peptide YVEEL and the HIV-1 TAT (48–60) hybrid peptide. Peptide was labeled with FITC. **(A)** MS Spectrums of the indicated peptides; **(B)** microscopy images of FHC cells treated with FITC-YVEEL (20 μM) for 20, 40, and 60 min, respectively; **(C)** peptide YVEEL was fused to HIV-1 TAT protein (48–60) and labeled with FITC [FITC-^Tat(48– 60)^ YVEEL]. Microscopy images of FHC cells treated with FITC-^Tat(48– 60)^ YVEEL (20 μM) for 20, 40, and 60 min, respectively; **(D)** microscopy images of FHC and IEC-6 cells treated with FITC-^Tat(48– 60)^ YVEEL for 1 h at × 400 magnification. Nuclei were stained with DAPI (blue) before imaging. Bar = 50 μm.

To elucidate the cellular uptake efficiency of the peptide, YVEEL was labeled with FITC (FITC-YVEEL) and incubated with FHC enterocytes under cell culture conditions. MS Spectrums of the synthetic peptides were shown in Figure 1A. Fluorescence microscopy was used to examine the internalization of FITC-YVEEL as early as 10 min after the incubation. However, faint cell-associated fluorescence was observed within 1 h incubation period (Figure 1B). To enhance the cell penetrating ability of YVEEL, the HIV-1 TAT (48–60)-hybrid YVEEL with FITC labeling [FITC-^Tat(48–60)^ YVEEL] was synthesized and the cellular uptake efficiency was evaluated. The FITC-^Tat(48–60)^ YVEEL showed a rapidly higher cellular uptake at 40 min of incubation (Figure 1C). In addition, FITC-^Tat(48–60)^ YVEEL displayed the predominant cytoplasmic and nuclear enrichment in both FHC and IEC-6 enterocytes (Figure 1D). These results demonstrated that fusion with HIV-1 TAT (48–60) could increase the internalization of YVEEL by enterocytes.

### Effects of ^Tat^ ^(48–60)^ YVEEL on toll-like receptor 4-mediated signaling and cell migration of enterocytes

**FIGURE 2:**
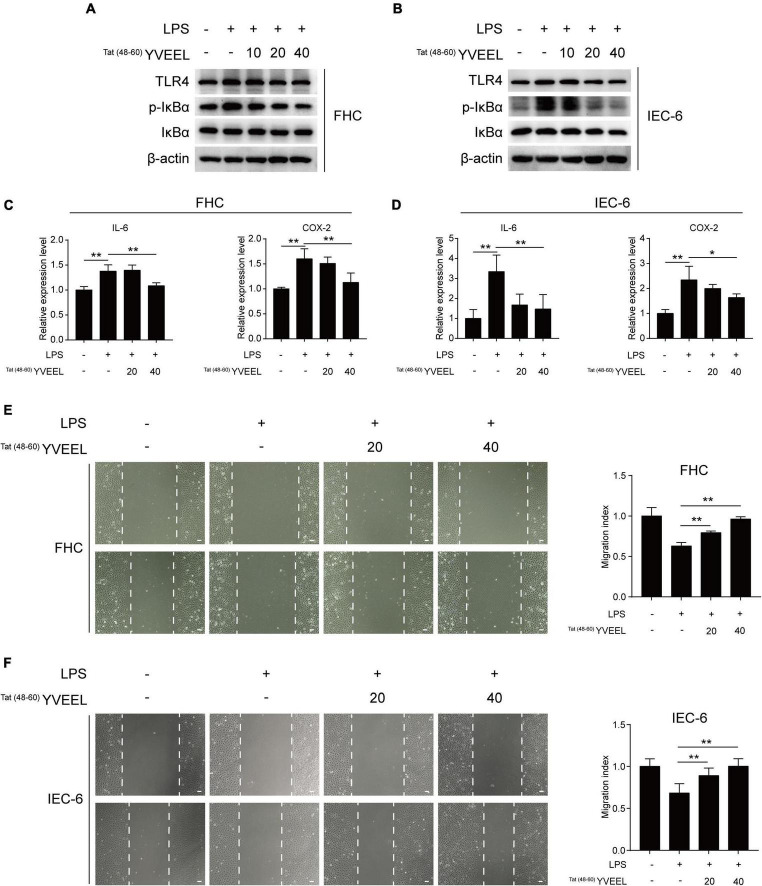
^Tat(48– 60)^ YVEEL inhibited TLR4-mediated signaling and restored cell migration of enterocytes. Cells were pretreated with ^Tat(48– 60)^ YVEEL at the indicated concentration (0, 10, 20, and 40 μM) for 1 h, followed by LPS treatment. Western blot analysis of TLR4 signaling in **(A)** FHC and **(B)** IEC-6 cells after 12 h LPS treatment; mRNA expression of IL-6 and COX-2 in **(C)** FHC and **(D)** IEC-6 cells treated with LPS for 6 h in the absence or presence of ^Tat(48– 60)^ YVEEL; cell migration after induction of mechanical scratch under ^Tat(48– 60)^ YVEEL treatment with LPS for 12 h in **(E)** FHC and for 16 h in **(F)** IEC-6 cells; bar = 50 μm, at × 200 magnification, the quantitative analyses of cell migration were shown in the right panel, respectively. *p < 0.05, **p < 0.01.

The critical role of LPS receptor Toll-like receptor 4 (TLR4) in the pathogenesis of NEC has been well documented (3). We first sought to determine the effect of ^Tat(48–60)^ YVEEL on TLR4-mediated signaling in enterocytes *in vitro*. FHC and IEC-6 enterocytes were treated with LPS, and the degree of TLR4-mediated signaling was measured by assessing the protein level of TLR4 and p-IκBα as well as the mRNA expression of the pro-inflammatory cytokine interleukin-6 (IL-6) and cyclooxygenase-2 (COX-2). Consistent with previous observations (23), LPS induced TLR4-mediated signaling in enterocytes. Treatment of enterocytes with ^Tat(48–60)^ YVEEL significantly reduced the protein level of TLR4 and p-IκBα (Figures 2A,B) as well as the IL-6 and COX-2 mRNA expression (Figures 2C,D). Previous studies have demonstrated that the activation of TLR4 inhibits enterocyte migration, reducing the capacity for intestinal repair and restitution, which contributed to NEC development (24). In view of this, we next investigated the effects of ^Tat(48–60)^ YVEEL on enterocyte migration. As shown in Figures 2E,F, the exposure of FHC and IEC-6 enterocytes to LPS led to a marked reduction in enterocyte migration, which was significantly restored in the presence of ^Tat(48–60)^ YVEEL.

### Protective effects of ^Tat(48–60)^ YVEEL is associated with phosphatidylinositol 3-kinase/AKT inhibition

**FIGURE 3:**
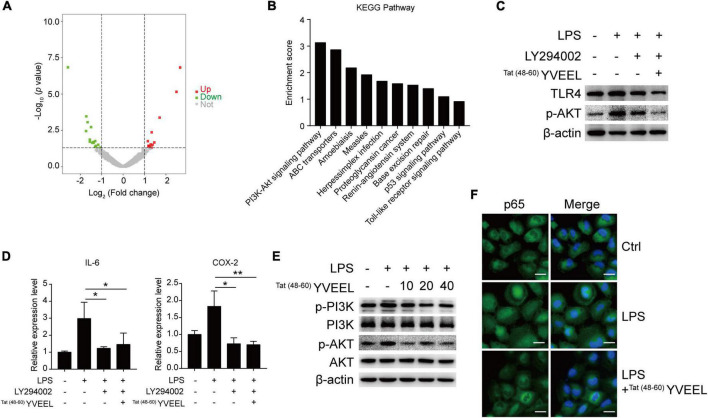
Phosphatidylinositol 3-kinase (PI3K)/AKT pathway was involved in the protective effects of ^Tat(48– 60)^ YVEEL in FHC cells. Cells were pretreated with ^Tat(48– 60)^ YVEEL at 40 μM for 1 h, followed by LPS treatment. **(A)** Volcano plot of genes differentially expressed in LPS and LPS + ^Tat(48– 60)^ YVEEL treated FHC cells, up- and downregulated genes with fold change ≥2.0 are highlighted in red and green, respectively; **(B)** genes with differential expression between LPS and LPS + ^Tat(48– 60)^ YVEEL treated FHC cells were subjected to KEGG pathway analysis. The top 10 most significantly KEGG pathways are shown in the graph; **(C)** the PI3K/AKT inhibitor LY294002 has similar effect as ^Tat(48– 60)^ YVEEL on LPS-induced TLR4 expression in FHC cells; **(D)** LY294002 has similar effect as ^Tat(48– 60)^ YVEEL on LPS-induced IL-6 and Cox-2 mRNA expression in FHC cells. *p < 0.05, **p < 0.01; **(E)** Western blot analysis showing the phosphorylation of PI3K and AKT in FHC cells treated with LPS and ^Tat(48– 60)^ YVEEL at the indicated concentration; **(F)** representative graphs of Control (Ctrl), LPS and LPS + ^Tat(48– 60)^ YVEEL treated FHC cells stained for the p65 subunit of NF-κB (green) and nuclei (DAPI, blue), at × 400 magnification, bar = 25 μm.

In order to understand how ^Tat(48–60)^ YVEEL regulates the TLR4-mediated signaling in enterocytes, RNA sequencing was performed to compare the differences in mRNA expression levels between LPS and LPS + ^Tat(48–60)^ YVEEL treated FHC enterocytes (Figure 3A). By Kyoto Encyclopedia of Genes and Genomes (KEGG) pathway analysis, we noticed that a subset of differentially expressed gene are related to the phosphatidylinositol 3-kinase (PI3K)/AKT pathway, suggesting that this pathway might be involved in the protective effects of ^Tat(48–60)^ YVEEL on TLR4-induced enterocyte dysfunction (Figure 3B). As shown in Figure 3C, the PI3K inhibitor LY294002 could inhibit TLR4 protein expression in FHC enterocytes, which was more significant in the presence of ^Tat(48–60)^ YVEEL. Moreover, TLR4-induced mRNA expression of IL-6 and COX-2 was also reduced upon LY294002 treatment in the absence or presence of ^Tat(48–60)^ YVEEL (Figure 3D). To further determine the effect of ^Tat(48–60)^ YVEEL on the PI3K/AKT pathway in enterocytes, FHC enterocytes were incubated with LPS, along with ^Tat(48–60)^ YVEEL, or not. The phosphorylation of PI3K and AKT was analyzed using western blotting. Our results revealed that LPS treatment significantly increased the phosphorylation of PI3K and AKT in enterocytes, which was markedly inhibited in the presence of ^Tat(48–60)^ YVEEL (Figure 3E). As shown in Figure 3F, the p65 subunit of nuclear factor-κB (NF-κB) was translocated from the cytoplasm to the nucleus under the stimulation of LPS. However, the TLR4-mediated p65 translocation was obviously blocked by ^Tat(48−60)^ YVEEL treatment. These data suggest that the inhibitory effects of ^Tat(48–60)^ YVEEL on TLR4-mediated signaling may be mediated *via* PI3K/AKT inhibition.

### ^Tat(48–60)^ YVEEL reversed toll-like receptor 4-mediated signaling in an phosphatidylinositol 3-kinase/AKT-dependent manner

**FIGURE 4:**
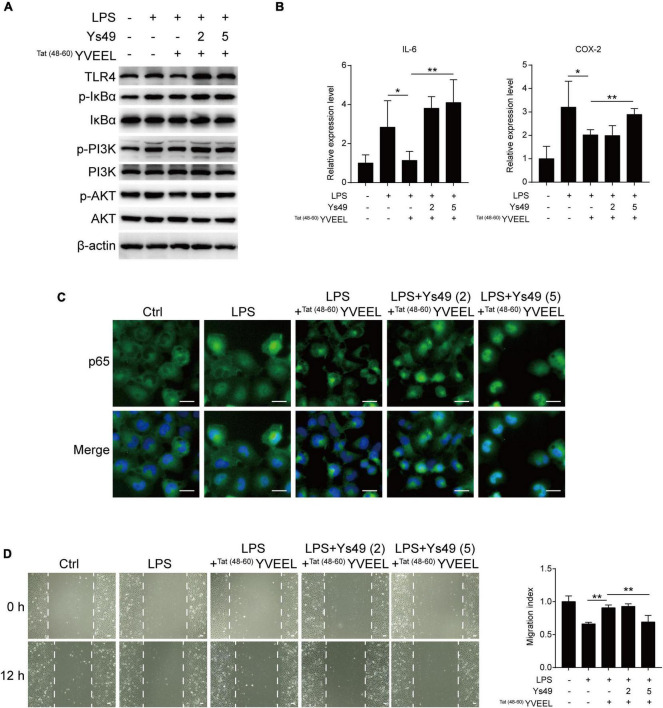
^Tat(48– 60)^ YVEEL attenuates TLR4-mediated signaling and restores cell migration *via* inhibiting PI3K/AKT pathway. Cells were pretreated with ^Tat(48– 60)^ YVEEL (40 μM) and the PI3K/AKT activator Ys49 (2 and 5 μM) for 1 h, followed by LPS treatment. **(A)** Western blot analysis showing the TLR4-mediated signaling and the phosphorylation of PI3K and AKT in FHC cells; **(B)** mRNA expression of IL-6 and COX-2 in FHC cells in each indicated group, *p < 0.05; **(C)** representative graphs of Control (Ctrl), LPS, LPS + ^Tat(48– 60)^ YVEEL and LPS + ^Tat(48– 60)^ YVEEL + Ys49 (2 and 5 μM) treated FHC cells stained for the p65 subunit of NF-κB (green) and nuclei (DAPI, blue), at × 400 magnification, bar = 25 μm; **(D)** cell migration after induction of mechanical scratch in FHC cells under indicated treatment, at × 200 magnification, bar = 50 μm, the quantitative analyses of cell migration were shown in the right panel. **p < 0.01.

To confirm the involvement of PI3K/AKT, we assessed the effect of the PI3K activator Ys49 on TLR4-mediated signaling in FHC enterocytes with or without ^Tat(48–60)^ YVEEL treatment. The PI3K activator Ys49 significantly abolished inhibitory effects of ^Tat(48–60)^ YVEEL on PI3K/AKT pathway (Figure 4A). Moreover, in enterocytes treated with Ys49, the ^Tat(48–60)^ YVEEL-induced reduction of TLR4 and p-IκBα expression was notably reversed (Figure 4A). Treatment of enterocytes with Ys49 also reversed the protective effect of ^Tat(48–60)^ YVEEL on TLR4-mediated induction of pro-inflammatory cytokines (Figure 4B). In addition, the blockage of TLR4-mediated cytoplasm-nucleus translocation of p65 by ^Tat(48−60)^ YVEEL (Figure 4C) and the protective effect of ^Tat(48–60)^ YVEEL on enterocyte migration were attenuated upon Ys49 treatment as well (Figure 4D). Taken together, these results illustrate that TLR4-mediated signaling in enterocytes is blocked by ^Tat(48–60)^ YVEEL through inhibition of PI3K/AKT pathway.

### ^Tat(48–60)^ YVEEL attenuates necrotizing enterocolitis severity in rat models

**FIGURE 5:**
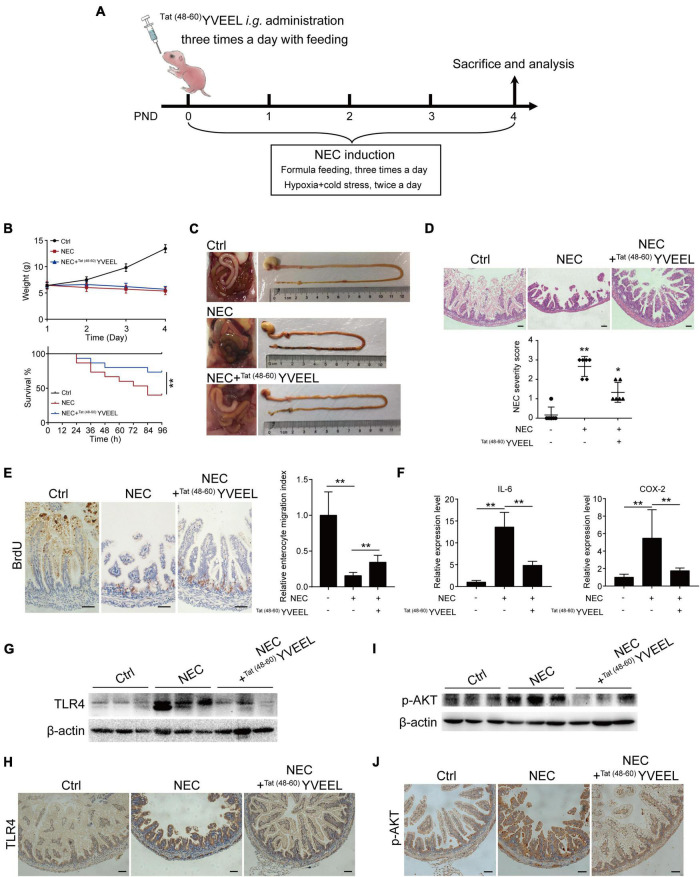
^Tat(48– 60)^ YVEEL promotes migration of enterocytes and reduces the severity of experimental NEC. **(A)** Experimental model of NEC–a timeline of treatment strategy. PND: postnatal day. **(B)** Body weight and survival rate of rat pups after experimental NEC with or without ^Tat(48– 60)^ YVEEL treatment; **(C)** representative photographs of the intestines of newborn mice that were mother fed (Ctrl), exposed to experimental NEC (NEC), or exposed to experimental NEC with daily oral gavage with ^Tat(48– 60)^ YVEEL at 10 mg/kg (NEC + ^Tat(48– 60)^ YVEEL); **(D)** representative photomicrographs of the terminal ileum of the indicated group, bar = 200 μm, quantification of NEC severity score in the group indicated were shown in the bottom, *p < 0.05, **p < 0.01; **(E)** pups in the indicated group were injected with BrdU 4 h before killing and immunostained with BrdU. Micrographs showing the migration of enterocytes in the terminal ileum of the indicated group, enterocyte migration index was quantified by measuring the ascent of BrdU-labeled enterocytes from the crypt to the villus, bar = 200 μm; **(F)** qRT-PCR of IL-6 and COX-2 in intestine of the pups in the indicated group, *p < 0.05, **p < 0.01; **(G)** Western blot analysis showing the TLR4 expression in intestine of the pups in each group; **(H)** TLR4 immunostaining was performed on the sections of terminal ileum in the indicated group, bar = 200 μm; **(I)** Western blot analysis showing the p-AKT expression in intestine of the pups in each group; **(J)** p-AKT immunostaining was performed on the sections of terminal ileum in the indicated group, bar = 200 μm.

To define whether ^Tat(48–60)^ YVEEL could attenuate the severity of NEC and, if so, whether PI3K/AKT pathway was involved, we subjected newborn rat pups to experimental NEC (Figure 5A) as previously described (20). Mother-fed pups (Ctrl) exhibited steady increases in body weight. Body weight and survival rate of NEC group gradually decreased throughout the experiment. There were no differences in body weight between NEC and NEC + ^Tat(48–60)^ YVEEL groups. However, the survival rate at 96 h increased from 40% in NEC group to 73.33% in NEC + ^Tat(48–60)^ YVEEL group (Figure 5B). NEC severity was significantly attenuated in ^Tat(48–60)^ YVEEL treated pups, as manifested by a reduction in the degree of gross pneumatosis intestinalis and intestinal ischemia (Figure 5C). H&E staining revealed the swollen and broken villi and disordered arrangement of lamina propria cells in NEC group. However, reduced microscopic intestinal mucosal injury and decreased pathological severity score were found in NEC + ^Tat(48–60)^ YVEEL group (Figure 5D). Enterocyte migration *in vivo* was determined by examining the extent of BrdU-labeled enterocyte migration as previously validated (3). Pups subjected to experimental NEC showed a significantly reduced extent of migration of BrdU-labeled enterocytes along the crypt-villus axis, that was partially restored by the administration of ^Tat(48–60)^ YVEEL (Figure 5E). Increased expression of the pro-inflammatory cytokine IL-6 and COX-2 within the intestine of NEC group was also inhibited by the ^Tat(48–60)^ YVEEL administration (Figure 5F). To assess the effects of ^Tat(48–60)^ YVEEL on *in vivo* TLR4 activation, protein level of TLR4 in the intestinal epithelium was determined by immunohistochemistry and western blotting. As shown in Figures 5G,H, pups subjected to experimental NEC showed an extremely high level of TLR4. By contrast, the supplementation of NEC pups with the ^Tat(48–60)^ YVEEL markedly reduced the TLR4 protein level. Importantly, ^Tat(48–60)^ YVEEL administration significantly inhibited the PI3K/AKT pathway in the intestines of NEC pups as revealed by a reduction in p-AKT protein level (Figures 5I,J). Together, these findings imply that ^Tat(48–60)^ YVEEL inhibits TLR4-mediated signaling in the newborn intestine through PI3K/AKT pathway.

### Liposome encapsulation enhances the bioavailability of ^Tat(48–60)^ YVEEL *in vivo*

**FIGURE 6:**
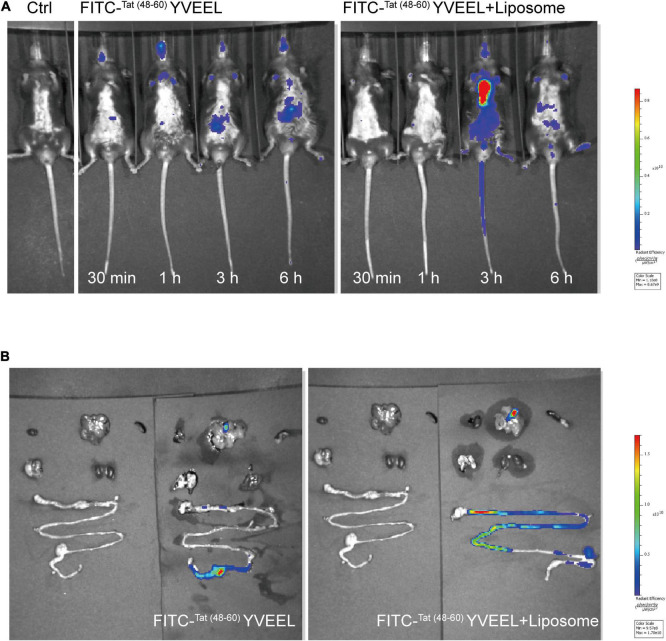
Liposome encapsulation enhances dissolution and prolongs the intestinal residence time of ^Tat(48– 60)^ YVEEL. **(A)** Distribution of FITC-^Tat(48– 60)^ YVEEL and FITC-^Tat(48– 60)^ YVEEL encapsulated into liposomes (FITC-^Tat(48– 60)^ YVEEL + liposomes) at indicated time after delivery of liposomes by oral gavage in C57BL6/J mice. **(B)** Fluorescence signal from FITC-^Tat(48– 60)^ YVEEL and FITC-^Tat(48– 60)^ YVEEL + liposomes, delivered through oral gavage in C57BL6/J mice 3 h after administration.

It has been reported that encapsulation of bioactive peptides into liposomes may offer a potential alternative way to enhance their stability and efficacy (25). In view of this, the *in vivo* behavior of free and liposome-encapsulated ^Tat(48–60)^ YVEEL was determined by using fluorescent-labeled ^Tat(48–60)^ YVEEL. Free and FITC-^Tat(48–60)^ YVEEL-loaded liposomes was orally gavage fed. As a result, mice administrated with FITC-^Tat(48–60)^ YVEEL-loaded liposomes shows stronger fluorescence signal than that administrated with free FITC-^Tat(48–60)^ YVEEL at 3 h after gavage (Figure 6A). Moreover, the liposome-encapsulated ^Tat(48–60)^ YVEEL showed a long retention of signal primarily in the intestines (Figure 6B), suggesting a promising approach to enhance the bioavailability and therapeutic effect of ^Tat(48–60)^ YVEEL on NEC.

## Discussion

Necrotizing enterocolitis is a devastating intestinal disorder marked by abnormal bacterial colonization, exaggerated inflammation and oxidative stress that affects preterm neonates (26). Because of the multifunctional properties such as anti-microbial, immunomodulatory, and anti-oxidant (27), bioactive peptides derived from milk protein are promising for the NEC treatment. Unfortunately, oral delivery of bioactive peptides faces great challenge of low bioavailability partially due to the gastrointestinal environment (28). In the previous studies, a whey-derived anti-oxidative peptide YVEEL has been found to enhance the proliferation and osteogenic differentiation of primary osteoblast cells *in vitro* (29) and exert osteoprotective potential by suppressing inflammatory status and inducing bone formation factors *in vivo* (17). However, the effect of YVEEL on NEC remains unknown to date. To enhance cellular uptake of YVEEL by intestinal epithelial cells, a cell-penetrating TAT modified peptide ^Tat(48–60)^ YVEEL was prepared in current study. It has been reported that cell penetrating peptides (CPPs), such as HIV-1 TAT (48–60), were capable of enhancing delivery and absorption of molecules through intestinal epithelium (30). Indeed, the microscopy results clearly illustrate that HIV-1 TAT (48–60) can significantly promote the cellular uptake efficiency of YVEEL in intestinal epithelial cells *in vitro*. In addition, liposome encapsulation can extend the retention of ^Tat(48–60)^ YVEEL with the intestinal epithelium *in vivo*. Besides CPPs and liposomes, other technologies such as acylation can also improve the effective delivery of peptides *via* oral route. For instance, rational acylation of intestinotrophic glucagon-like peptide-2 (GLP-2) increases intestinal absorption and may enable oral administration, which could benefit patient compliance, in particular for chronic disorders locally in the gastrointestinal tract such as Crohn’s (31). More efforts are needed to further improve the bioavailability of ^Tat(48–60)^ YVEEL in the future.

Here, we show that ^Tat(48–60)^ YVEEL has an ability to reduce the severity of experimental NEC *via* inhibiting TLR4-mediated signaling within intestinal epithelium. Specifically, activation of TLR4-mediated signaling results in exaggerated inflammation and impaired enterocyte migration for intestinal repair, which was significantly ameliorated after ^Tat(48–60)^ YVEEL administration. Emerging evidence suggests that increased expression of TLR4 in the intestinal epithelium a key contributor to the pathogenesis of NEC (32). TLR4 could activate nuclear factor-κB (NF-κB) signaling, further inducing the production of inflammatory cytokines (e.g., IL-6, COX-2 and TNF-α) and downstream inflammatory responses (33). The expression level of inflammatory cytokines such as IL-6 was correlated with the severity of NEC (34). Previous studies have been indicated that higher IL-6 levels were detected in the plasma and stools of NEC patients in the follow-up (35). Moreover, IL-6 levels were corrected with disease severity and can be used to monitoring the progress of NEC (36, 37). COX-2 is an important factor in gut homeostasis and inflammation. High levels of COX-2 were also observed in NEC model and in human NEC (38, 39). In addition, the expression of IL-6 and COX-2 were both regulated by TLR4/NF-κB signaling, contributing to epithelial barrier integrity (40). In addition to the roles of TLR4 in regulation of inflammatory response, recent studies also implicate TLR4 activation in the regulation of enterocyte migration, goblet cell differentiation and lymphocyte influx during NEC. Autophagy induction in response to TLR4-mediated signaling is largely responsible for the impaired enterocyte migration, which results in the impairment in mucosal healing and intestinal restitution (24). Epithelial-specific TLR4 regulates goblet cell differentiation through Notch signaling pathway involved in the pathogenesis of NEC (4). Intestinal epithelial TLR4 activation alters the balance of tolerogenic Tregs vs. injurious Th17 cells in a STAT3-dependent manner, leading to the severe intestinal inflammation (41). Additionally, activation of endothelial TLR4 leads to reduction in intestinal perfusion *via* reduced eNOS signaling (6). TLR4 expressed in intestinal stem cells regulates their proliferation and apoptosis *via* activation of PUMA, which plays a critical role in the development of NEC (42). Therefore, further studies will be required in order to elucidate the comprehensive role of ^Tat(48–60)^ YVEEL in NEC prevention.

**FIGURE 7:**
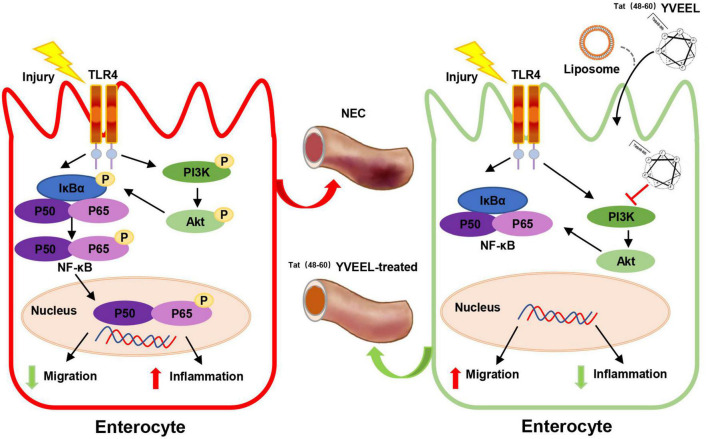
Scheme of the protective effects of ^Tat(48– 60)^ YVEEL on experimental NEC. ^Tat(48– 60)^ YVEEL exerts a powerful effect on TLR4-mediated signaling in enterocytes which involves the inhibition of PI3K/AKT pathway.

In seeking to define the mechanism involved, we found that these effects of ^Tat(48–60)^ YVEEL are dependent on PI3K/AKT signaling, as treatment with PI3K inhibitor LY294002 had similar protective effects that were abrogated with PI3K activator Ys49. The link between stimulation of TLR4 and PI3K/AKT activation in enterocyte inflammation and migration has been found by several groups. For instance, downregulating the PI3K/AKT pathway by berberine significantly reduces levels of inflammatory cytokines TNF-α and IL-6 in the epithelial cells of a NEC mouse model (43). Qureshi et al. demonstrate that endotoxin increases integrin expression in enterocytes and inhibits enterocyte migration by activating PI3K/AKT pathway, leading to impaired intestinal restitution and the development of NEC (44). In addition, endotoxin induces RhoA activation in a PI3K/AKT-dependent manner, which may be involved in the impaired enterocyte migration observed in experimental NEC (45). Our results demonstrated that PI3K/AKT pathway was activated in experimental NEC, and ^Tat(48−60)^ YVEEL significantly reduced the expression of pro-inflammatory cytokines, blocked the p65 nuclear translocation and restored the enterocyte migration by inhibiting the activation of PI3K/AKT pathway (Figure 7). Consistent with our findings, a recent study reveals that a hybrid peptide (LTP) by combining the native anti-inflammatory peptides (LL-37 and TP5) can alleviate LPS-induced damage in jejunum tissues. The anti-inflammatory mechanism of LTP was also associated with inhibition of TLR4 mediated NF-κB signaling and the phosphorylation of AKT (46).

## Conclusion

Taken together, we have now described a HIV-1 TAT (48–60)-hybrid peptide YVEEL with enhanced bioavailability. Importantly, ^Tat(48–60)^ YVEEL had protective effects on experimental NEC both *in vitro* and *in vivo*, *via* inhibiting TLR4-mediated signaling in a PI3K/AKT-dependent manner. The present findings provide new insight in developing novel therapeutic agents for NEC.

## Data availability statement

The data presented in the study are deposited in the NCBI GEO repository, accession number GSE214736.

## Ethics statement

This animal study was reviewed and approved by the Institutional Review Board at Women’s Hospital of Nanjing Medical University, Nanjing Maternity and Child Health Care Hospital.

## Author contributions

XC and SH designed the research. XY, WC, QY, and YCh performed the research. SY and CJ analyzed the data. YCa wrote the manuscript. All authors contributed to the article and approved the submitted version.

## Abbreviations

NEC: necrotizing enterocolitis
TLR4: toll-like receptor 4
LPS: lipopolysaccharides
2 ′ -FL: 2 ′ -fucosyllactose
6 ′ -SL: 6 ′ -sialyllactose
HPLC: high-performance liquid chromatography
FITC: fluorescein isothiocyanate
Ctrl: control
NF- κ B: nuclear factor- κ B
H&E: hematoxylin and eosin staining
IHC: Immunohistochemical
IL-6: interleukin-6
COX-2: cyclooxygenase-2
KEGG: Kyoto Encyclopedia of Genes and Genomes
PI3K: phosphatidylinositol 3-kinase
BrdU: bromodeoxyuridine
CPPs: cell penetrating peptides
GLP-2: glucagon-like peptide-2.
