# Supplementary material for: Peptide Tat(48–60) YVEEL protects against necrotizing enterocolitis through inhibition of toll-like receptor 4-mediated signaling in a phosphatidylinositol 3-kinase/AKT dependent manner
Source: Front Nutr. 2022 Oct 10;9:992145. doi: 10.3389/fnut.2022.992145 (PMC9590307; doi:10.3389/fnut.2022.992145)
Supplement: Supplementary file 1 [file Data_Sheet_1.pdf]

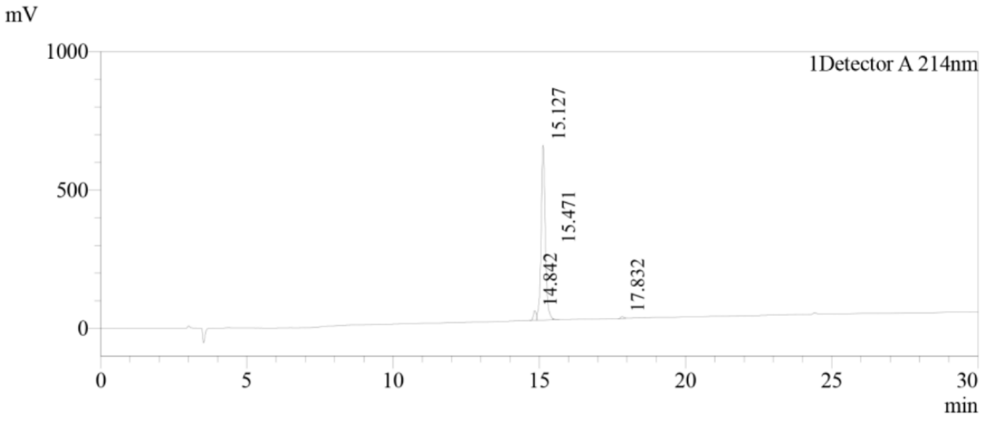

Peak Table

Detector A 214nm

Peak#	Ret. Time	Area	Height	Area%
1	14.842	231489	34905	3.518
2	15.127	6295154	632338	95.657
3	15.471	14672	4017	0.223
4	17.832	39684	6702	0.603
Total		6580999	677962	100.000

Supplementary Figure 1. The HPLC chromatogram of $^{Tat\ (48\text{-}60)}$ YVEEL. The purification yielded a final product with 95.657% purity.
